# Effects of red glasswort as sodium chloride substitute on the physicochemical properties of pork loin ham

**DOI:** 10.5713/ajas.19.0193

**Published:** 2019-07-01

**Authors:** Tae-Jun Jeong, Tae-Kyung Kim, Hyun-Wook Kim, Yun-Sang Choi

**Affiliations:** 1 Department of Food Science and Biotechnology of Animal Resources, Konkuk University, Seoul, 05029, Korea; 2 Research Group of Food Processing, Korea Food Research Institute, Wanju 55365, Korea; 3 Department of Animal Science and Biotechnology, Gyeongnam National University of Science and Technology, Jinju 52725, Korea

**Keywords:** Red Glasswort, Cooked Pork Loin Ham, Reduced Salt, Textural Properties

## Abstract

**Objective:**

This study was conducted to evaluate the effect of red glasswort (RG) (*Salicornia herbacea* L.) curing on the physicochemical, textural and sensory properties of cooked pork loin ham (*M. longissimus thoracis et lumborum*).

**Methods:**

All treatments were cured with different salt and RG powder levels. RG0 treatment was prepared with only 4% NaCl (w/w) as a control, and RG25, 3% NaCl:1% RG (w/w); RG50, 2% NaCl:2% RG (w/w); RG75, 1% NaCl:3% RG (w/w); RG100, 0% NaCl:4% RG (w/w) treatments were prepared sequentially. All samples were individually vacuum packaged in polyethylene bags and stored for 7 d at 3°C±1°C.

**Results:**

The results showed that as the rate of RG substitution increased, pH value, redness, myofibrillar protein solubility, and myofibrillar fragmentation index increased (p<0.05), but salt concentration and shear force decreased (p<0.05). However, there were no significant differences in cooking loss and moisture content. In terms of sensory evaluation, RG100 exhibited higher scores in tenderness and juiciness than RG0 (p<0.05).

**Conclusion:**

The partial substitution of NaCl by RG could improve the physicochemical properties, textural and sensory characteristics of cooked pork loin. Therefore, it is suggested that RG as a natural salt replacer could be an effective ingredient for developing low-sodium cured hams.

## INTRODUCTION

Sodium chloride (NaCl) is one of the main ingredients for manufacturing meat products [1]. Generally, the effects of sodium chloride on meat products can be classified into three functions. Firstly, salt plays an important role in producing desirable texture. This is related to an increase in myofibril solubility, which contributes to the improvement of water holding capacity, resulting in the texture formation of meat products. Secondly, salt confers enhancing the flavor of meat products by perceived saltiness. Lastly, salt in meat products is responsible for shelf stability. Salt makes to reduce water activity and affects osmotic pressure on microorganisms [1,2].

Although salts have essential functionalities in processed meats, many organizations ef fort to reduce NaCl content. Excessive sodium intake causes increased blood pressure and leads to cardiovascular diseases [3–5]. Salt could be harmful when consumed more than 5 g per day. This is equivalent to 2 g Na/100 g per day (WHO, 2016). Low-salt meat products can easily be prepared by reducing salt content. However, a reduction in salt below a certain level results in adverse effects, including defective textures due to excess of proteolysis as well as the decline of shelf stability. In addition, the salt reduction of meat products could lead to unpleasant flavors by overacting polypeptides, resulting from the excessive content of low molecular weight nitrogen compounds such as peptides and free amino acids [6].

Numerous studies have attempted to prepare salt mixtures via partial substitution of NaCl by KCl, CaCl_2_, or MgCl_2_ and natural ingredients such as seaweeds [7,8]. Although these strategies could reduce the content of Na in meat products, they revealed problems of bitterness and metallic flavors [3].

Glasswort ( *Salicornia herbacea* L.) is a halophyte, well-known as Hamcho in Korea, that naturally grows on the Korean mudflat. Kim et al [9] reported that red glasswort (RG) contains 9.09% sodium, 1.31% potassium, 0.83% calcium, 0.49% magnesium, and 69.24% total dietary fiber. The several compounds found in RG contribute to beneficial impacts on human health [10]. In this regard, glasswort has been extensively applied as a natural source for developing the functionalities of several food products including soybean curd [11], brown sauce [12], rice cake [13], and kimchi [14].

The objective of this study was to evaluate the effects of RG as a NaCl substitute on the physicochemical, textural and sensory properties of cooked pork loin ham (*M. longissimus thoracis et lumborum*).

## MATERIALS AND METHODS

### Preparation of red glasswort powders

Freeze-dried RG (*Salicornia herbacea* L.) powder was purchased from a local market. The RG used in this study was harvested in Sinan (Korea), including the whole part of RG. The detail information of RG powder used in this study was previously mentioned by Kim et al [9]; the RG powder contains pH value (5.79), Commission Internationale de l’Eclairage (CIE) L* (61.43), CIE a* (0.76), CIE b* (17.01), sodium (8.09% [w/w]), potassium (1.31% [w/w]), calcium (0.83% [w/w]), magnesium (0.49% [w/w]), and total dietary fiber (69.24% [w/w]) [9].

### Preparation of cured pork loin

Twenty fresh pork loins (*M. longissimus thoracis et lumborum*, n = 20) were obtained from a local meat market and randomly divided into five groups. Each pork loin of group cut into steak size with approximate thickness of 2.54 cm and weight of 100 g. Each steak sample (40 steaks each treatment) was cured with salt and RG mixtures by rubbing. RG0 group was salted with 4% (w/w) NaCl content (100% NaCl). The other groups were prepared by partially substituting NaCl with RG. The RG25 was salted with 3% NaCl and 1% RG (w/w), the RG50 was salted with 2% NaCl and 2% RG (w/w), the RG75 was salted with 1% NaCl and 3% RG (w/w), and finally, RG100 was salted with only 4% RG (w/w). Briefly, the ratios of NaCl and RG were given in Table 1. After the process, salted samples were individually vacuum-packaged in polyethylene bags and stored for 7 days in a refrigerator (3°C±1°C).

### pH measurements

The pH values were measured by sampling 5 g of cooked sample and homogenizing with 20 mL distilled water for 60 s at 8,000 rpm speed (Ultra-Turrax T25, Janke & Kunkel, Staufen im Breisgau, Germany). The pH value of the cooked sample was determined using an electronic pH meter (Model 340, Mettler-Toledo GmbH, Greifensee, Swizerland).

### Color measurement

The color measurements of samples were conducted after cooking. The color values of core sections from each cooked sample were measured using a colorimeter (Minolta Chroma meter CR-210, Konica Minolta, Tokyo, Japan) with a CIE L* (lightness), CIE a* (redness), and CIE b* (yellowness). Before color measurement, the colorimeter was calibrated with a white plate (CIE L* = +97.83, CIE a* = −0.43, CIE b* = +1.98). Ten readings were obtained for each sample.

### Moisture content

The moisture content of samples was determined. Briefly, three grams of sample was dried in a drying oven at 105°C during 12 h. Moisture content was calculated by the percentage weight difference between before drying weight and after drying weight.

### Sensory evaluation

The sensory evaluation of samples was conducted by trained panelists. The preliminary sensory tests using various meat products were performed several times for their training. Each cooked sample was cut into 1.5×1.5×2.54 cm^3^ pieces and served to the panelists in random order with a three-digit code. The score results of each sample were obtained in terms of color, flavor, tenderness, juiciness, salinity, off-flavor, and overall acceptability. The color, flavor, off-flavor, and overall acceptability (1 = extremely undesirable, 10 = extremely desirable), tenderness and juiciness (1 = extremely tough, 10 = extremely tender), salinity (1 = extremely unsalted, 10 = extremely salty) of the cooked samples were determined by using a 10 point scale.

### Salt concentration

Five grams of cooked samples were homogenized with 45 mL of distilled water by using Ultra-Turrax T25 at 8,000 rpm for 60 s (Janke & Kunkel, Germany). The salt concentration of homogenates was measured by using a salinometer (TM-30D, Takemura Electric Works Ltd., Tokyo, Japan).

### Cooking loss

After curing for 7 days, the cured meats were removed from polyethylene bags, and the unabsorbed salt and RG powder on the surface of sample were eliminated with paper towels. After sealing again in a polyethylene bag, each sample was cooked at 80°C until the core temperature reached at 72°C. After cooking, the sample was cooled at room temperature (25°C) for 2 h and reweighed. The cooking loss (%) of samples was calculated as follows:

Cooking loss (%)=[weight of sample before cooking (g)-weight of sample after cooking (g)]/weight of sample before cooking (g)×100

### Myofibril fragmentation index

The myofibril fragmentation index (MFI) was conducted by the procedure of Kim et al [9] with slight modification. The uncooked samples of cured meat (4 g) were mixed with 40 mL of MFI buffer solution (20 mM K_2_HPO_4_/KH_2_PO_4_ buffer, pH 7.0 with 100 mN KCl, 1 mM ethylenediaminetetraacetic acid, 1 mM NaN_3_) by using a homogenizer. The homogenate was centrifuged at 1,000 *g* for 15 min (4°C). The supernatant of sample was decanted and carried out three times above steps. The third supernatant was removed, mixed again with MFI buffer, and then filtered using a strainer. The protein concentration of suspended myofibril in MFI solution was determined by Biuret method and adjusted to 0.5 mg/mL [9]. The MFI values were calculated as follows; MFI = an absorbance at 540 nm×200.

### Warner-Bratzler shear force

The Warner-Bratzler shear force (WBSF) of cooked samples was obtained with 1.25 cm of cores by using a sampling device. The WBSF was recorded from the maximum force (kg) by using TA-XT2*i* (Stable Micro Systems Ltd., Surrey, England) equipped with a V-type blade. The speed condition of the WBSF measurement was designed at 5 mm/s (pre-test and test speed) and 10 mm/s (post-test speed).

### Protein solubility

The protein solubility was conducted by using the modified method of Kim et al [9]. The uncooked sample of cured meat (2 g) was mixed with 20 mL (sarcoplasmic protein solubility buffer: ice-cold 0.025 M potassium phosphate buffer at pH 7.2 and total protein solubility buffer: 20 mL 1.1 M potassium iodide in 0.1 M potassium phosphate buffer pH 7.2) by using a homogenizer and homogenized samples were left overnight at 4°C. On the next day, the samples were centrifuged at 6,000 *g* for 20 min and the protein concentration of supernatant was measured by using the Biuret method. Salt-soluble myofibrillar protein solubility was calculated by difference between total and sarcoplasmic protein solubility.

### Sodium dodecyl sulfate-polyacrylamide gel electrophoresis

The sodium dodecyl sulfate-polyacrylamide gel electrophoresis (SDS-PAGE) was conducted by using the modified method of Li et al [15]. After the protein concentration of the samples were set to 0.5 mg protein/mL by the method of Biuret and composition of gel was 12% running gels and 5% stacking gels (Bio-Rad Lab, Inc, St. Louis, MO, USA). The loaded gel was stained with Coomassie Brilliant Blue (B7920, Sigma, St. Louis, MO, USA), and destaining buffer were prepared with acetic acid, distilled water and methanol (10:40:50). The molecular weight of separated protein bands was identified by comparing with those of standard protein markers (Precision Plus Protein Standards, Catalog number 1610374, Bio-Rad Lab., USA).

### Statistical analysis

The experimental design was completely randomized. Three independent replicates were conducted for each experiment. Data were analyzed using SPSS version 20. (SPSS Inc., Chicago, IL, USA) by one-way analysis of variance to determine the significance of main effect (treatment). Duncan’s multiple range test (p<0.05) was used to determine differences between means.

## RESULTS AND DISCUSSION

### Physicochemical properties

The effects of substitution of NaCl by RG on the physicochemical characteristics of cooked pork loins are shown in Table 2. The pH values of the cooked pork loins ranged from 5.72 (RG0) to 5.77 (RG100). The gap in pH was numerically small, but there was a significant difference in pH value among treatments (p<0.05). Previously, Kim et al [9] reported that substitution of NaCl with RG (0 % to 1%) led to small changes in the pH value of cooked sausage in the range of 6.13 to 6.16. In this study, the substitution of RG seemed to have little effect on the pH (pH 5.79) of cooked pork loins.

The substitution of NaCl with RG affected CIE L* (lightness) and a* (redness) of cooked pork loin (Table 2). The lightness and redness values of cooked pork loins treated with RG powder were higher than those of RG0 (p<0.05), whereas the yellowness was similar for all treatments (p>0.05). The lightness was significantly different among treatments, but with small numerical differences. With increasing RG content, the redness was obviously increased from 4.83 (RG0) to 7.21 (RG100) (p<0.05). This was likely due to pigments in RG, such as chlorophyll a and chlorophyll b. In general, carotenoid component in glasswort is increased in the autumn, resulting in a color change from green to flushed red [16]. In turn, chlorophyll b and carotenoid pigments might be responsible for the red color of cooked pork loins.

The moisture content ranged from 66.5% to 67.5% with no significant difference among all treatments (p>0.05). Previously, Ruusunen et al [17] reported that water-holding capacity could decrease with reducing salt content from 1.7% to 1.3%. However, the result of this current study showed no change in moisture content despite decreased salt content. This phenomenon may be associated with dietary fiber and salt contained in RG.

### Sensory evaluation

The effects of RG substitution with RG on the sensory properties of cooked pork loins are presented in Table 3. The salinity of pork loins was decreased with increasing the substitution level of NaCl with RG (p<0.05). This result might be related to relatively low salt content of RG. There were no significant (p>0.05) differences in the flavor and off-flavor scores. In terms of overall acceptability, RG100 and RG0 (control) showed similar scores (p>0.05), but RG100 had the highest score (p<0.05). RG100 also obtained the highest scores in tenderness and juiciness (p<0.05). Jiménez-Colmenero et al [7] reported that frankfurters containing 1% NaCl and 1% seaweed received similar scores in texture acceptability compared to control (2% NaCl). In a previous study, Kim et al [9] reported that RGs contained a total dietary fiber content of 69.24%. The dietary fiber in RG could be responsible for the enhancement of water binding properties. Therefore, the increased scores of tenderness and juiciness due to the substitution of NaCl with RG might be greatly related to the dietary fibers contained in RG.

### Salt concentration

**Figure 1:**
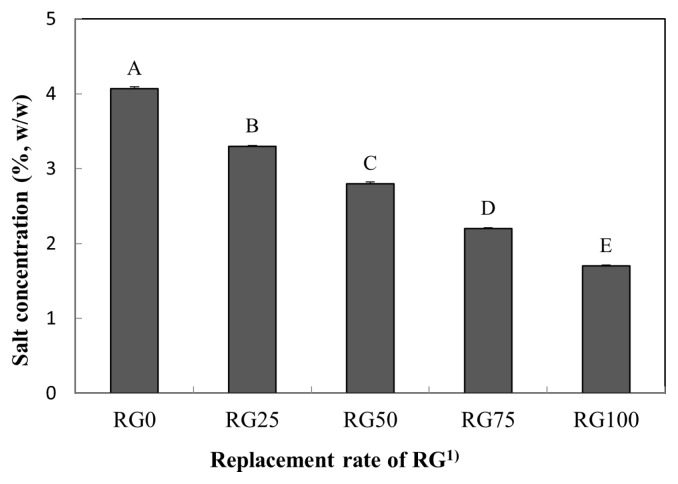
Substituting effect of NaCl by red glasswort (RG) on salt concentration in cooked pork loin hams. ^1)^ RG0 (control), 4% NaCl; RG25, 3% NaCl and 1% RG; RG50, 2% NaCl and 2% RG; RG75, 1% NaCl and 3% RG; RG100, 4% RG. ^A–E^ Means with different letters are different (p<0.05).

The effects of NaCl substitution by RG on the salt concentration in pork loins are shown in Figure 1. RG0, RG25, RG50, RG75, and RG100 had salt concentrations of 4.07%, 3.30%, 2.80%, 2.20%, and 1.70%, respectively. As expected, an increase in the RG level evidently decreased the salt concentration of pork loins (p<0.05). The salt concentration could be influenced by the level of RG, since the RG used in this study contained approximately 0.25% salt. As a result, RG100 showed the lowest salt concentration among all treatments (p<0.05).

### Cooking loss

**Figure 2:**
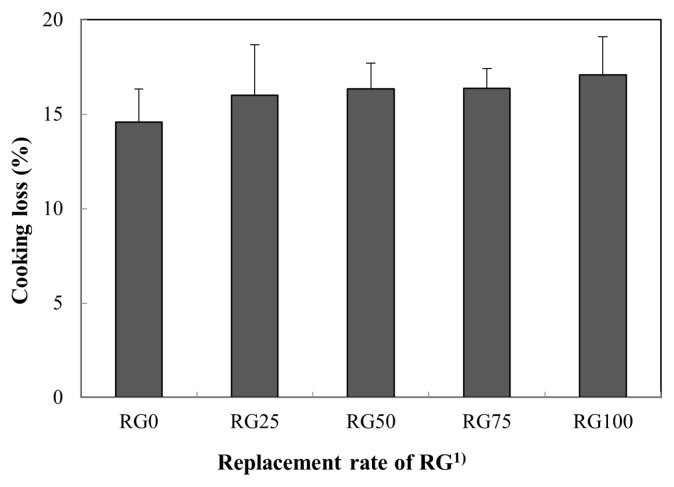
Substituting effect of NaCl by red glasswort (RG) on cooking losss in cooked pork loin hams. ^1)^ RG0 (control), 4% NaCl; RG25, 3% NaCl and 1% RG; RG50, 2% NaCl and 2% RG; RG75, 1% NaCl and 3% RG; RG100, 4% RG.

The effect of NaCl substitution by RG on cooking loss of pork loins is shown in Figure 2. Although a difference in salt concentration between RG0 and RG100 was found, there was no significant difference in cooking loss (p>0.05), indicating that the dietary fibers in RG positively improved the cooking loss. Salt concentration is an important factor affecting cooking loss, as demonstrated by the decrease in cooking loss when the salt concentration was increased from 0% to 1% [18]. Kim et al [19] indicated that chicken cured with 1% salt had a greater cooking loss (at approximately 14.8%) compared to chicken cured with 3% salt. Ruusunen et al [18] reported that ham cooked with 1.1% salt presented a greater cooking loss (at approximately 30.9%) compared ham cooked with 2.6% salt. Given the results of previous studies, despite reduced salt concentration with the replacement of NaCl with RG, no difference in cooking loss of cooked pork loins could be attributed to the water-binding capacity of RG.

### Myofibril fragmentation index and shear force

**Figure 3:**
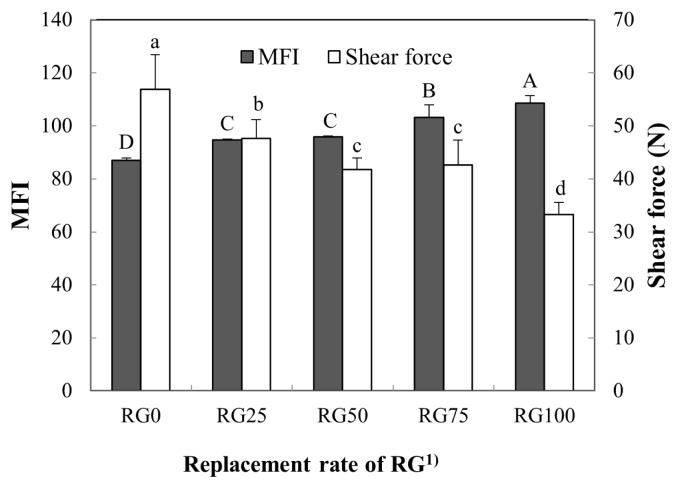
Substituting effect of NaCl by red glasswort (RG) on myofibrillar fragementation index (MFI) and shear force of pork loin. ^1)^ RG0 (control), 4% NaCl; RG25, 3% NaCl and 1% RG; RG50, 2% NaCl and 2% RG; RG75, 1% NaCl and 3% RG; RG100, 4% RG. ^A–D, a–d^ Means with different letters are different (p<0.05).

The MFI and shear force of pork loins salted with replacing NaCl with RG are shown in Figure 3. The MFI of pork loins was increased by the increase in RG level, and RG100 showed the highest MFI among all treatments (p<0.05); an increase of approximately 25% compared to the RG0 treatment. The z-lines of muscle structure can be destroyed by proteolysis of myofibril proteins. Degradation of myofibril proteins by endogenous proteases and calcium, magnesium, and zinc chloride leads to the fragmentation of myofibrils and results in increased MFI [20,21]. Glasswort exerts protease activity [22] and including minerals such as potassium, calcium, and magnesium [9,23]. Nuruk made of glasswort has higher potassium and magnesium contents than that without glasswort. These results indicated that RG could have protease activity and/or potassium minerals, which might lead to increased myofibrillar proteolysis.

The shear force of pork loins was decreased by increasing RG level. RG25, RG50, RG75, and RG100 showed 16%, 26%, 25%, and 41% lower shear force values than RG0 (control) (p<0.05). In previous studies, shear force could be negatively correlated with MFI [24]. Thus, high MFI is an excellent predictor for the tenderness of loin muscles. In this study, RG100 showed the lowest shear force among all treatments (p<0.05). This result indicates that an increase in RG could cause the proteolysis of myofibrillar protein, as determined by MFI, which could in turn reduce the shear force of cooked pork loins.

### Protein solubility

**Figure 4:**
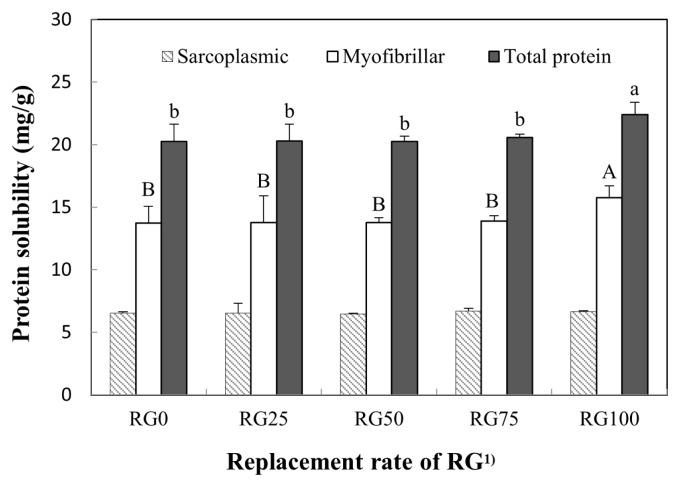
Substituting effect of NaCl by red glasswort (RG) on protein solubility in cured pork loins. ^1)^ RG0 (control), 4% NaCl; RG25, 3% NaCl and 1% RG; RG50, 2% NaCl and 2% RG; RG75, 1% NaCl and 3% RG; RG100, 4% RG. ^A,B,a,b^ Means with different letters are different (p<0.05).

The effect of replacing NaCl with RG on the protein solubility (total, myofibrillar, and sarcoplasmic protein) of pork loins is shown in Figure 4. The amount of RG added is directly correlated with myofibril protein solubility (p<0.05). However, the sarcoplasmic protein solubility of cured pork loin was not affected by increasing the ratio of RG (p>0.05). RG100 had the highest total and myofibril protein solubility among all treatments (p<0.05). The solubility of proteins is largely related to the tenderizing effect, especially myofibrillar protein solubility [25]. Generally, myofibril protein solubility is affected by salt concentration. Previously, Kim et al [9] reported that the addition of 1% RG in frankfurters increased the myofibril protein solubility, but had no difference in the water soluble protein solubility. Munasinghe and Sakai [26] reported that the conformational change in myosin, one of major myofibril proteins, is differently affected by lithium, sodium, and potassium ions. Furthermore, Nayak et al [20] reported that 0.05% CaCl_2_ and MgCl_2_ caused the highest myofibril protein solubility among all treatments. In this study, thus, the potassium and calcium contained in RG may affect the total and myofibril protein solubility of RG100.

### Sodium dodecyl sulfate-polyacrylamide gel electrophoresis

**Figure 5:**
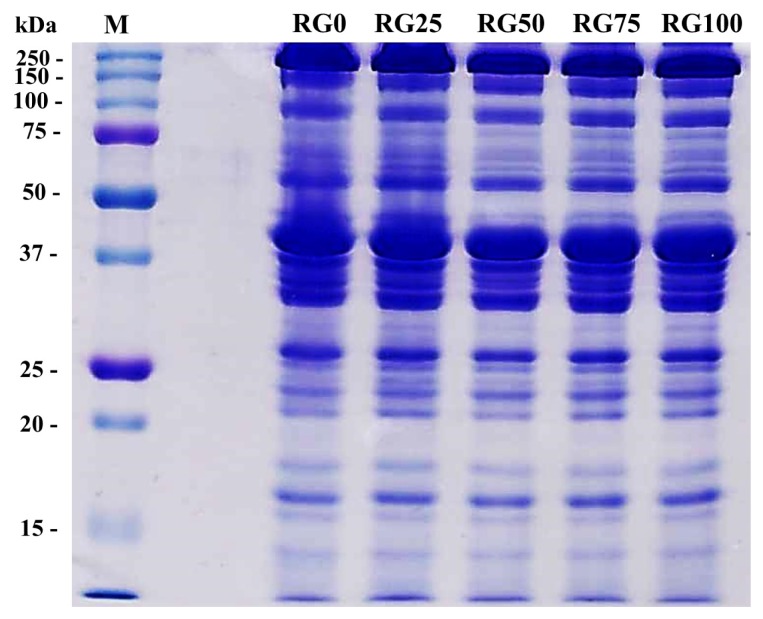
Sodium dodecyl sulfate-polyacrylamide gel electrophoresis (SDS-PAGE) Photographs of myofibrils extracted from pork loins cured with the different ratio between NaCl and red glasswort (RG). RG0 (control), 4% NaCl; RG25, 3% NaCl and 1% RG; RG50, 2% NaCl and 2% RG; RG75, 1% NaCl and 3% RG; RG100, 4% RG.

The SDS-PAGE was performed to observe protein patterns of pork loins treated with RG (Figure 5). Hwang et al [27] reported that the increase in MFI was related to the degradation of troponin T and z-disk by calcium-activated factor, as a result, a 30-kDa protein appeared. In this study, however, the appearance of a 30-kDa protein was not observed despite the increased MFI. Therefore, the protein bands revealed by SDS-PAGE were insufficient to observe specific degradation of troponin T. Similarly, Sonaiya et al [28] reported no visual difference in the protein degradation patterns of beef muscles, while increased MFI was found. Sams et al [29] reported that there were no consistent differences in protein band patterns in SDS-PAGE system, although there were minor changes in myofibril structure.

## CONCLUSION

In conclusion, the replacement of NaCl with RG evidently decreased the salt concentration of cooked pork loin. The salt concentration of RG100 was approximately 58% lower compared to that of RG0. The shear force of RG100 treatment was approximately 41% lower than that of RG0, despite no impacts on moisture content and cooking loss. The decrease in shear force might be due to protease activity and the high content of minerals in RG. For these reasons, pork loins cured with RG had higher MFI. Consequently, RG100 showed improvements in physicochemical properties and sensory attributes related to tenderness and juiciness of cooked pork loins. Therefore, RG could be used as a potentially favorable natural salt substitute to improve the textural properties in pork loins, together with a reduction in salt concentration.

## Figures and Tables

**Table 1:** Percentage formula (%) of sodium chloride and red glasswort (RG) in cooked pork loin hams

Traits	Replacement rate of RG1)				

	RG0	RG25	RG50	RG75	RG100
Sodium chloride (%)	4	3	2	1	-
RG (%)	-	1	2	3	4

1) RG0 (control), 4% NaCl; RG25, 3% NaCl and 1% RG; RG50, 2% NaCl and 2% RG; RG75, 1% NaCl and 3% RG; RG100, 4% RG.

**Table 2:** Substituting effect of NaCl by red glasswort (RG) on pH, color and moisture content of cooked pork loin hams

Traits	Replacement rate of RG1)					SEM

	RG0	RG25	RG50	RG75	RG100	
pH value	5.72^c^	5.72^c^	5.76^b^	5.76^b^	5.77^a^	0.004
CIE L*-value	71.94^b^	71.57^b^	73.06^ab^	74.63^a^	74.84^a^	0.440
CIE a*-value	4.83^b^	5.49^b^	5.49^b^	7.13^a^	7.21^a^	0.233
CIE b*-value	7.71	6.83	7.29	7.14	7.01	0.128
Moisture (%)	66.98	66.92	67.57	66.55	67.46	0.295

SEM, standard error of the means; CIE, Commission Internationale de l’Eclairage.

1) RG0 (control), 4% NaCl; RG25, 3% NaCl and 1% RG; RG50, 2% NaCl and 2% RG; RG75, 1% NaCl and 3% RG; RG100, 4% RG.

a–c Means within a row with different letters among treatments are significantly different (p<0.05).

**Table 3:** Substituting effect of NaCl by red glasswort (RG) on sensory properties of cooked pork loin hams

Traits	Replacement rate of RG1)					SEM

	RG0	RG25	RG50	RG75	RG100	
Color	6.8^b^	6.8^b^	6.9^b^	7.5^a^	7.5^a^	0.128
Flavor	7.3	6.8	6.9	7.0	7.0	0.121
Tenderness	5.9^bc^	5.3^c^	5.7^c^	7.0^ab^	7.3^a^	0.209
Juiciness	5.8^bc^	5.4^c^	6.0abc	7.0^ab^	7.2^a^	0.211
Salinity	8.0^a^	5.8^a^	5.6^a^	4.5^b^	3.5^c^	0.248
Off-flavor	3.4	3.6	3.4	3.6	3.8	0.320
Overall acceptability	7.0^ab^	6.3^b^	6.7^b^	7.1^ab^	7.8^a^	0.144

SEM, standard error of the means.

1) RG0 (control), 4% NaCl; RG25, 3% NaCl and 1% RG; RG50, 2% NaCl and 2% RG; RG75, 1% NaCl and 3% RG; RG100, 4% RG.

a–c Means within a row with different letters among treatments are significantly different (p<0.05).
